# Biomarker Identification and Effect Estimation on Schizophrenia – A High Dimensional Data Analysis

**DOI:** 10.3389/fpubh.2015.00075

**Published:** 2015-05-05

**Authors:** Yuanzhang Li, Robert Yolken, David N. Cowan, Michael R. Boivin, Tianqing Liu, David W. Niebuhr

**Affiliations:** ^1^Preventive Medicine Branch, Walter Reed Army Institute of Research (WRAIR), Silver Spring, MD, USA; ^2^Department of Pediatrics, Johns Hopkins University School of Medicine, Baltimore, MD, USA; ^3^ManTech International Corporation, Health and Life Sciences, Herndon, VA, USA; ^4^Department of Statistics, George Washington University, Washington, DC, USA; ^5^Division of Epidemiology and Biostatistics, Department of Preventive Medicine and Biometrics, Uniformed Services University of the Health Sciences, Bethesda, MD, USA

**Keywords:** schizophrenia, biomarker identification, space decomposition, gradient, high dimensional data

## Abstract

Biomarkers have been examined in schizophrenia research for decades. Medical morbidity and mortality rates, as well as personal and societal costs, are associated with schizophrenia patients. The identification of biomarkers and alleles, which often have a small effect individually, may help to develop new diagnostic tests for early identification and treatment. Currently, there is not a commonly accepted statistical approach to identify predictive biomarkers from high dimensional data. We used space decomposition-gradient-regression (DGR) method to select biomarkers, which are associated with the risk of schizophrenia. Then, we used the gradient scores, generated from the selected biomarkers, as the prediction factor in regression to estimate their effects. We also used an alternative approach, classification and regression tree, to compare the biomarker selected by DGR and found about 70% of the selected biomarkers were the same. However, the advantage of DGR is that it can evaluate individual effects for each biomarker from their combined effect. In DGR analysis of serum specimens of US military service members with a diagnosis of schizophrenia from 1992 to 2005 and their controls, Alpha-1-Antitrypsin (AAT), Interleukin-6 receptor (IL-6r) and connective tissue growth factor were selected to identify schizophrenia for males; and AAT, Apolipoprotein B and Sortilin were selected for females. If these findings from military subjects are replicated by other studies, they suggest the possibility of a novel biomarker panel as an adjunct to earlier diagnosis and initiation of treatment.

## Introduction

The development of objective tools facilitating identification of subjects at high risk or in a prodromal stage of schizophrenia could enable early interventions aimed to prevent disease occurrence or improve disease course and consequently reduce the healthcare costs. The precise etiology of schizophrenia remains uncertain and is most likely multifactorial and complex. Although there is a great body of research elucidating various factors associated with schizophrenia, some of these results are inconsistent. Thus, schizophrenia etiology is highly unlikely to be limited to a single risk factor whether genetic, epigenetic, or environmental. The pathogenesis of schizophrenia involves dysfunction of immune, endocrine, and central nervous systems and multiple corresponding qualitative and quantitative biochemical alterations (1). Some of these changes could be measured in sera and used to identify individuals with prodromal disease. Because serum level variation of a single biochemical molecule might have a very small effect size, we identified, measured, and analyzed a combination of 48 potential biomarkers.

The complexity of the task is compounded by the heterogeneity of schizophrenia reflected in a broad variety of its clinical presentations, some of which could be a result of different etiopathogenic pathways (2). Studies to determine the most common biochemical variations distinguishing those who will develop schizophrenia from those who will not, would be difficult to replicate in different schizophrenia populations.

Detecting multiple biomarkers with small individual effects requires large sample sizes, a large number of biomarkers, and appropriate statistical approaches to ensure that valuable information is not lost. Regression of high dimensional data is difficult for at least two reasons: sample size and collinearity. When the sample size is small, traditional regression methods that use the sample covariance, such as the ordinary least squares (OLS) approach, perform poorly (3). Therefore, using predictive modeling for multivariate regression with a large number of biomarkers and other possible explanatory/predictive variables, for the process of variable selection and dimension reduction is very challenging. Multicollinearity, a high correlation of two or more predictors in a multiple regression, may lead to erratic changes in the effects of individual biomarkers and large SEs of the coefficient estimates in response to small changes in the model. As a result it makes the selection of biomarkers difficult, because the estimated effect of the predictor variables is expected to be biased. A high degree of multicollinearity also leads to either software failure in matrix inversion or inaccurate results.

Principal component analysis (PCA) and ridge regression (4) are commonly used to solve the collinearities in regression. PCA is also a common method used to reduce the number of predictive variables. But PCA identifies linear combinations of variables to summarize the data in the process. It does not use information of the dependent variable for the construction of these linear combinations. The first principal component is often not the linear combination of the input variables that is most significantly associated with the dependent variable of disease state (5, 6). The ridge regression has the same difficulty as OLS when the sample size is small.

Decision tree learning is a method commonly used in data mining (7), which is a model that predicts the value of a target variable based on several input variables. The two main types of analyses are: classification tree analysis and regression tree analysis. Classification and regression tree (CART) analysis uses both of the above procedures, first introduced by Breiman et al (8).

In this study, we applied a decomposition-gradient-regression (DGR) method, which was originally introduced by Li and Niebuhr in 2012 Joint Statistical Meeting (http://www.amstat.org/meetings/jsm/2012/onlineprogram/AbstractDetails.cfm?abstractid=304618), to select biomarkers for identify the risk of schizophrenia from a high dimensional case-control data set. The first step in DGR is to separate the correlated biomarkers into several subspaces; second step is to find gradient and its orthogonal vectors in each subspace; and finally, to perform multiple linear regression on the gradients from each subspace is performed. The biomarkers in each subspace are independent, and it does not violate the assumptions of regression modeling, and we can select the biomarkers used in regression without collinearity effects. We also compare the results of this approach with the CART method in this study.

## Methods

### Data

Data for US military service members who received medical discharges with a diagnosis of schizophrenia from 1992 to 2005 were obtained from the US Army Physical Disability Agency, the Secretary of the Navy Council of Review Boards, and the Air Force Personnel Center/US Air Force Physical Disability Division (9). Those aged 18 and older who were on active duty at the time of their schizophrenia diagnosis, and who had at least one serum sample of 0.5 ml or greater in the Department of Defense Serum Repository (DoDSR) obtained before diagnosis were selected as potential study cases. Nearly all (99%) study subjects were hospitalized with a psychiatric disorder before their discharge from military service. The time of schizophrenia onset was estimated as the earliest date of either the first hospitalization with psychiatric disorder International Classification of Disease 9th Revision (ICD-9-CM) codes (290-319) or the date the medical or physical evaluation boards was initiated.

Control subjects were selected from the active duty US military service population who were over the age of 18 and had no inpatient or outpatient mental health diagnoses. All control subjects were matched to their cases on gender, race, branch of military service, date of birth (±12 months), and military enlistment (±12 months).

The medical and demographic data from 1989 to 2006 were provided in 2007 by the Defense Medical Surveillance System, Armed Forces Health Surveillance Center (AFHSC), US. Department of Defense (DoD), Silver Spring, MD. Serum specimens from 1988 to 2006 were retrieved in 2007 from the Department of Defense Serum Repository (DoDSR), AFHSC, US. DoD, Silver Spring, MD. However, due to budget limitations, only a subset of all the schizophrenia cases with their matched controls was selected for serum sample retrieval. Serum specimens were originally collected every 2 years for HIV screening and stored at −30°F. At least one, and up to four, matched (±90 days) specimens were selected for each study subject. The time of specimen collection for controls was determined by date of collection of their matched cases. The 48 potential biomarkers used in this study are listed in (see Table 1). The subject distribution and sample distribution are shown in (see Table 2).

**Table 1:** **List of biomarkers for schizophrenia after decomposition by subspaces of observed collinearity**.

**Subspace A**
Testosterone, Total
Prolactin (PRL)
Interleukin-6 receptor (IL-6r)
Brain-derived neurotrophic factor (BDNF)
Follicle-stimulating hormone (FSH)
Fetuin-A
Apolipoprotein A-I (Apo A-I)
Interleukin-7 (IL-7)
Carcinoembryonic antigen (CEA)
Beta-2-Microglobulin (B2M)
Prostatic acid phosphatase (PAP)
Peptide YY (PYY)
Macrophage migration inhibitory factor (MIF)
Epidermal growth factor receptor (EGFR)
Serum amyloid P-component (SAP)
Vascular endothelial growth factor (VEGF)
Immunoglobulin M (IGM)
TNF-related apoptosis-inducing ligand
Receptor 3 (TRAIL-R3)
Interleukin-10 (IL-10)
Luteinizing hormone (LH)
Matrix Metalloproteinase-2 (MMP-2)
Vitronectin
Endothelin-1 (ET-1)
CD5 (CD5L)
Alpha-1-antitrypsin (AAT)
Apolipoprotein B (Apo B)
Macrophage-derived chemokine (MDC)
Cortisol (Cortisol)
Ferritin (FRTN)
Intercellular adhesion molecule 1 (ICAM-1)
Betacellulin (BTC)
Cancer antigen 125 (CA-125)
Monocyte chemotactic protein 2 (MCP-2)
**Subspace B**
Apolipoprotein H (Apo H)
Tumor necrosis factor receptor 2 (TNFR2)
Connective tissue growth factor (CTGF)
Sortilin
Kidney Injury Molecule-1 (KIM-1)
Macrophage Inflammatory Protein-1 alpha (MIP-1 alpha)
Serotransferrin (Transferrin)
Thyroid-stimulating hormone (TSH)
Apolipoprotein C-I (Apo C-I)
Haptoglobin
Tissue inhibitor of metalloproteinases 1 (TIMP-1)
Immunoglobulin A (IgA)
**Subspace C**
Apolipoprotein A-II (Apo A-II)
Complement C3 (C3)
Calbindin

**Table 2:** **Subject and serum specimen frequencies by gender, race, and age**.

Factor	Level	Schizophrenia subjects		Serum specimens	
		Count	%	Count	%
Gender	Female	25	8.5	65	12.2
	Male	269	91.5	469	87.8
Race	Black	90	30.6	170	31.8
	Other	31	10.5	60	11.2
	White	173	58.8	304	56.9
Age	<25	191	65.0	349	65.4
	≥25	103	35.0	185	34.6

### Statistical analysis

Multicollinearity generates biased estimation in multiple linear regressions. To avoid collinearity, we first separated the correlated biomarkers into different groups, which we called subspaces. Second, we found the gradient direction which is the normal vector of a hyperplane in each subspace that best separates the cases and controls within the subspace (10). The gradient score is the linear combination of the standardized values of biomarkers used in each subspace. Scores were generated for the gradient and their perpendicular vectors in each subspace. The gradient score and the other significant vector scores from each subspace were used as the factor in statistical modeling. Third, we eliminated the noneffect biomarkers backwards by examining the coefficients of the gradient and the effect of gradient score modeling in each subspace. Then applying the regression model on the gradient scores of select biomarkers, we evaluated the joint effect of biomarkers on schizophrenia and the individual effect for schizophrenia.

Two kinds of data were used to check the effects of the gradient and the orthogonal vector scores. One is the US military data, and the other is simulated data, which includes one binary outcome Y and 100 predictors. In the simulated data, a few predictors have effect on Y with selected association, while the others have no effects on Y. The results from both kinds of data showed that no score other than the gradient score had a significant effect to distinguish the binary outcome. The gradient score consisted of nearly all the information from all biomarkers in the gradient.

Given that multiple serum samples were collected for each subject from different times before diagnosis, the generalized estimating equation was used to estimate the unknown parameters (11). The odds ratio and 95% confidence interval or *p*-value was reported using Bonferroni correction. The degrees of freedom of the Wald chi-square value of the gradient score was adjusted by the number of biomarkers used in the gradient vector.

The coefficient of a biomarker in the gradient vector describes its contribution to distinguish the schizophrenia cases from the controls. If the coefficient of a given biomarker is near 0, it implies that it has no effect on schizophrenia, which can then be eliminated from the gradient without loss of information. Hence, we can eliminate the nonsignificant biomarkers one by one.

Two approaches for the number of biomarkers to be selected were used. The first approach was decided by the surface of sensitivity versus the numbers of biomarkers used in subspaces A and B constructed by regression model for all subspaces with different dimensions or by biological plausibility arguments from epidemiologists. The second approach used the Akaike information criterion (AIC): AIC = 2p − 2Log L, which is a measure of the quality of fit of a statistical model for a given set of data, where *p* is the number of parameters in the regression. For longitudinal GEE regression, the quasi-likelihood information criterion (QIC), which uses the quasi-likelihood to replace the likelihood in AIC, is commonly used (12). When we use the gradient score in the model, we count the number of parameters in the gradient score as *k* rather than 1, where *k* is the number of biomarkers used in the gradient score. It can be seen from (see Table 2), the military data was longitudinal, and hence we minimized the adjusted QIC for selection of the final predictive biomarkers.

## Results

### Biomarker selection

Among the 48 biomarkers, one pair was highly correlated, with a Pearson correlation coefficient of 0.87; three pairs had an absolute value of Pearson correlation coefficient over 0.6, and over 20 pairs of biomarkers, had an absolute value of Pearson correlation coefficients over 0.40. First, using Pearson correlation coefficient of ±0.4 as the threshold, the 48 biomarkers were assigned to three groups: subspaces A, B, and C. There were three biomarkers in subspace C; all of them were eliminated because they made no contribution to identify schizophrenia cases. The effect of gradient C score was around 0. The gradient scores were highly significant in both subspaces A and B by using Bonferroni criterion (adjusted *p* < 0.05/k_A_ and 0.05/k_B_, respectively, where k_A_ and k_B_ were the number of biomarkers used in the gradients in subspaces A and B).

**Figure 1:**
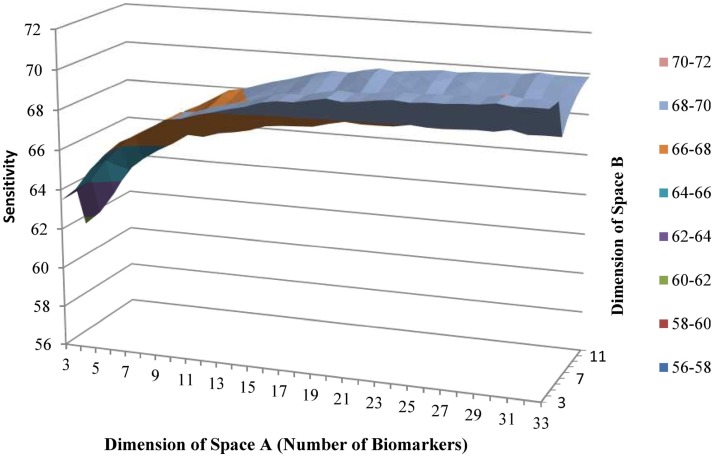
**Average sensitivity to predict schizophrenia of training group by number of biomarkers in subspaces A and B**.

**Figure 2:**
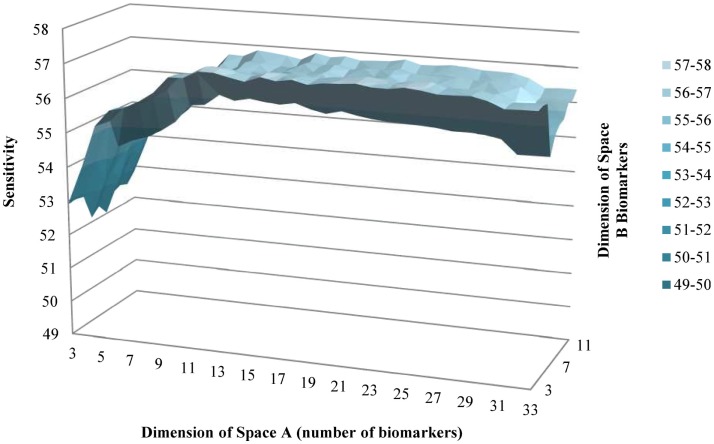
**Average sensitivity to predict schizophrenia of testing group by number of biomarkers in subspaces A and B**.

**Figure 3:**
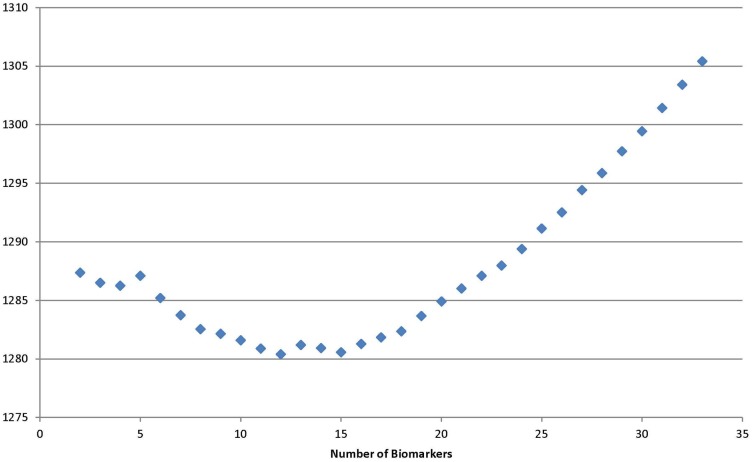
**Quasi-likelihood information criterion in subspace A**.

**Figure 4:**
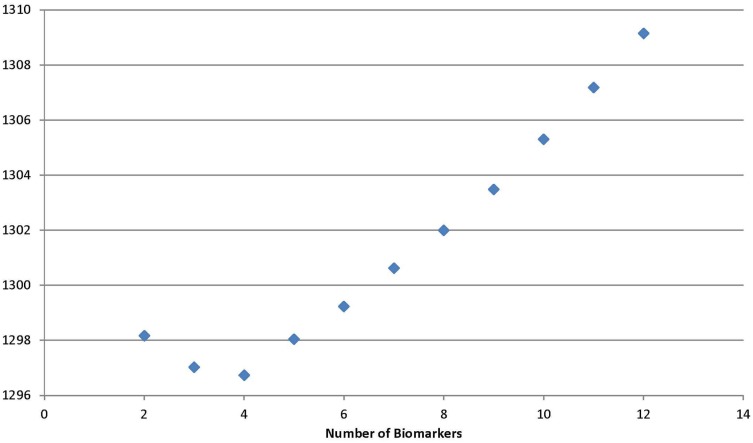
**Quasi-likelihood information criterion in subspace B**.

Using the backward selection approach, the biomarker with the coefficient nearest to 0 in the gradient was eliminated one by one. The average sensitivity for schizophrenia status among 100 simulations was used to make the surface graphs by the number of biomarker used in subspaces A and B (see Figures 1 and 2). For each simulation, two-thirds of subjects were selected randomly in the training dataset to fit the model, and the remaining one-third of the subjects was in the testing set to verify the model. For the subspace A, the sensitivity increased with the number of biomarkers until peaks for the training set (13 biomarkers) and testing set (11 biomarkers) were reached. Further increases in the number of biomarkers did not yield any improvement in sensitivity. Selecting four biomarkers for subspace B was sufficient as no monotonic change in sensitivity was observed when the number was further increased. The QIC curve by the number of biomarkers in subspace A is shown in (Figure 3), which was minimized at *k* = 12. But, the curve has a jump at five and six biomarkers. For subspace B in Figure 4, three and four biomarkers were the optimal selection.

### The effects of selected biomarkers

Next, we used the selected 12 and 6 biomarkers from subspace A as well as three biomarkers from subspace B to generate the gradient scores to fit the logistic model with a GEE approach. We found only minor difference between odds ratios in the two situations of different number of biomarkers used in the subspace A. The OR = 1.77 for increasing one SD of gradient score using 12 biomarkers, and OR = 1.5 for the gradient increasing using six more significant biomarkers among the 12; both were significant. This implies that six additional biomarkers made minor contributions in distinguishing schizophrenia cases from controls. The results for six biomarkers in subspace A and three biomarkers in subspace B are shown in Table 3. The gradient was the most significant vector among the orthogonal vectors in subspace A, and the *p*-value was much smaller than the critical value by Bonferroni correction (0.0083) for both males and females. Similarly for the three biomarkers in subspace B, by adjusting for multiple comparisons (α = 0.0167) the gradient score was also significant. More conservatively, by using Wald chi-square approach, the adjusted chi-squared test *p*-value for gradient score in subspace A was still highly significant, while the *p*-value for gradient score in subspace B only approached significance (13).

**Table 3:** **The odds ratio for one SD increasing in gradient score**.

Gender	Gradient score by subspace	OR	ORL	ORU	Unadjusted *p*-value generated in model	Significance by Bonferroni correction	Adjusted Wald Chi-square *p*-value^a^
Female	A	3.88	2.29	7.03	2.10E–06	Yes	0.001
	B	1.82	1.2	2.86	0.00728	Yes	0.063
Male	A	1.5	1.27	1.77	2.00E–06	Yes	0.001
	B	1.25	1.05	1.5	0.01305	Yes	0.111
Both	A	1.79	1.51	2.15	7.87E–11	Yes	0
	B	1.19	1.05	1.36	0.006	Yes	0.074

*^a^Adjusted the degrees of freedom based on the number of biomarkers used in the gradient*.

Table 4 lists the standard coefficients of the biomarkers in the gradient scores. The percentage of contribution on the gradient score effect on schizophrenia is the square of the coefficients. The third column is the odds ratio for increasing one unit of the individual biomarkers along the gradient direction. Using the SE of the gradient score, the odds ratios of Alpha-1-antitrypsin (AAT), Interleukin-6 receptor (IL-6r) and connective tissue growth factor (CTGF) showed significant effects to identify schizophrenia for males; AAT, Apolipoprotein B (Apo B) and Sortilin showed significant effects for females. It was observed that the risks for increasing gradient score for females were much higher than that for males.

**Table 4:** **The risk of schizophrenia for selected biomarkers**.

Subspace	Male				Female			
	Biomarkers	Gradient score coefficient	Percentage contribution	Odds ratio^b^	Biomarker	Gradient score coefficient	Percentage contribution	Odds ratio^b^
A	Alpha-1-antitrypsin (AAT)	0.47	0.22	1.21^a^	Alpha-1-antitrypsin (AAT)	−0.58	0.34	0.45^a^
	Apolipoprotein A-I (Apo A-I)	0.38	0.14	1.16	Apolipoprotein B (Apo B)	0.48	0.23	1.92^a^
	Immunoglobulin M (IGM)	−0.33	0.11	0.87	Cortisol (Cortisol)	−0.30	0.09	0.66
	Interleukin-6 receptor (IL-6r)	−0.53	0.28	0.81^a^	Endothelin-1 (ET-1)	−0.33	0.11	0.64
	Prolactin (PRL)	0.36	0.13	1.16	Fetuin-A	0.36	0.13	1.62
	Serum amyloid P-component (SAP)	0.35	0.12	1.15	Interleukin-10 (IL-10)	0.32	0.10	1.54
B	Apolipoprotein H (Apo H)	−0.65	0.19	0.94	Haptoglobin	0.42	0.18	1.29
	Immunoglobulin A (IgA)	0.51	0.26	1.12	Sortilin	−0.73	0.54	0.64^a^
	Connective tissue growth factor (CTGF)	0.56	0.54	1.24^a^	Macrophage inflammatory protein-1 alpha (MIP-1 alpha)	−0.53	0.34	0.68

*^a^Significant at level <0.05*.

*^b^For one increasing SD*.

Table 5 shows biomarkers selected by DGR and CART methods; about 70% are the same. However, using CART, we cannot estimate the effect on the outcome by individual biomarkers.

**Table 5:** **List of biomarkers for schizophrenia selected by subspace using decomposition-gradient-regression method (DGR) versus classification and regression tree (CART)**.

Subspace	Biomarkers	
	DGR	CART
A	Alpha-1-antitrypsin (AAT)^a^	Alpha-1-antitrypsin (AAT)^a^
	Apolipoprotein A-I (Apo A-I)^a^	Brain-derived neurotrophic factor (BDNF)
	Epidermal growth factor receptor (EGFR)	Cancer antigen 125 (Ca-125)
	Ferritin (FRTN)	Carcinoembryonic antigen (Cea)
	Fetuin-A	Connective tissue growth factor (CTGF)^a^
	Immunoglobulin M (IGM)^a^	Cortisol (Cortisol)
	Interleukin-6 receptor (IL-6r)^a^	Interleukin-6 Receptor (IL-6r)^a^
	Macrophage migration inhibitory factor (MIF)^a^	Prostatic acid phosphatase (Pap)
	Peptide YY (PYY)	Sortilin^a^
	Prolactin (PRL)^a^	Macrophage migration inhibitory factor (Mif)^a^
	Serum amyloid P-Component (SAP)	Haptoglobin^a^
B	Apolipoprotein H (Apo H)	Immunoglobulin A (IgA)^a^
	Connective tissue growth factor (CTGF)^a^	Immunoglobulin M (IGM)^a^
	Haptoglobin^a^	Prolactin (PRL)^a^
	Immunoglobulin A (IgA)^a^	Thyroid-stimulating hormone (Tsh)
	Macrophage inflammatory protein-1 alpha (MIP-1 alpha)	Vitronectin
	Sortilin^a^	Apolipoprotein A-I (Apo A-I)^a^

*^a^Biomarkers are associated with decreased risk of schizophrenia by both DGR and CART*.

### Individual effect of biomarkers on schizophrenia

AAT was selected for both males and females by DGR, and had nearly the highest absolute effect for both genders, but was a risk factor for males and a protective factor for females. Similar results have been reported elsewhere (14). The signs and symptoms of schizophrenia and the age at which they appear, vary among individuals. In our data, the mean and SD of AAT for females were 21.1 (37.8) for cases, and 12.2 (17.3) for controls; for males, 7.8 (7.3) for cases and 6.5 (4.4) for controls. This explained the variation of the AAT effect by gender. Rudduck et al. studied the AAT effect on schizophrenia and claimed that significant differences with respect to phenotype (*p* < 0.05) and gene (*p* < 0.025) frequencies were found between the two groups of patients (15).

Interleukin contributed the highest risk (28%) in the gradient for males, as deficiency of one unit along the gradient direction increased the risk of schizophrenia by about 25% (OR = 0.81). Our findings were consistent with a recent study by Hope et al. (16).

The CTGF also showed a significant effect on schizophrenia status for males, with the highest contribution (54%) on the gradient score in subspace B. CTGF was identified to be associated with schizophrenia syndrome (17). Apo B was found to be a significant biomarker for risk of violent behavior (18).

The gradient score risk in subspace A for females was almost fourfold; the highest contribution was from AAT (34%), while Apo-B had the second highest contribution (24%). In prior studies, AAT was found to be correlated with violent behavior (19). Sortilin had the highest contribution (54%) on the gradient score for females in subspace B. However it was protective, with an OR of 0.64. Decreasing Sortilin would increase the risk of schizophrenia. We did not find literature on Sortilin’s effect on schizophrenia, however Chen et al., concluded that Sortilin was associated with BDNF (20). Similar to our findings, Reichelta and Landmark had found that IgA antibody increased schizophrenia risk (21). Apolipoprotein A-I (Apo A-I) was another risk factor of schizophrenia for males. An increase of one unit of Apo A-I in the gradient direction resulted in a nearly 16% increase in the risk of schizophrenia. It contributed 14% of the risk in the gradient. A recent study by Song et al. (22) suggested that APO A-I might be a novel biomarker related to metabolic side effects in first episode schizophrenia treated with risperidone (22). Another selected biomarker was prolactin, which contributed 13% of the risk and an odds ratio of 1.16, but not significant. This finding is consistent with Song et al. (23) finding that the schizophrenia group had higher serum levels of PRL, IL-1β, IL-6, and TNF-α compared with the control group (23). Wang et al. also concluded prolactin had an effect on schizophrenia status (24).

## Discussion

In this study, we validated a novel, three step biomarker selection processes to identify schizophrenia cases based on biological and technical reproducibility of the molecular signature. The first step involved decomposition of the sample space by examining the dependency of 48 biomarkers separated into three subspaces to avoid collinearity. The second step involved biomarker selection using the gradient in each subspace to use a few parameters in the regression without losing information and to select important biomarkers. Step three identified the effects of biomarkers’ in combination and individually. Comparable results were found by DGR and CART. The advantage of DGR is that the magnitude and direction of the biomarker effects can be estimated. The meaning of the association expressed as an odds ratio is also clearer and more easily explained in DGR.

We have used a novel approach to identify the potential biomarkers for diagnosis of schizophrenia. The reliable identification of biomarkers with predictive power among the high dimensional data is a key discipline in modern pharmaceutical and biotechnical research. Once these biomarkers have been found, we can use them to identify patients earlier and distinguish diseased from normal subjects. Selecting a few predictive biomarkers has a number of advantages in both epidemiology and statistical analyses: (1) the risk of over fitting is reduced which improves the predictive accuracy; (2) the number of parameters is reduced which decreases the sample collection costs; and (3) models based on fewer factors are often easier to interpret. The ideal biomarker selection method should achieve two objectives: eliminate trivial variables, and include whole groups of correlated predictors into the model, to identify biomarkers with strong joint effects (25). The DGR approach first separates the whole space into several subspaces. The correlated biomarkers with similar effects on prediction are separated into different subspaces; if one is selected in a subspace, usually the others will be selected in different subspaces. Due to multicollinearity, without decomposition, some or all will not be eliminated in the multiple regression. By using AIC/QIC or sensitivity analysis, we can decide the number of biomarkers to be selected: explainable with fewer predictors. We can set the statistical analytic program to select biomarkers automatically.

From the military data and the 48 biomarkers, AAT, Interleukin-6 receptor (IL-6r), and CTGF show significant effects to identify schizophrenia status in males; AAT, Apo B, and Sortilin show significant effects in females.

However, due to variations in serum specimens and the biomarker assays, some biomarkers might be selected by chance; the final selected biomarkers will likely vary from study to study. Ideally, the biological mechanisms of the biomarkers should be considered to avoid selection bias in the final predictive model. If these findings are replicated by other studies or data from other populations, they suggest the possibility of a novel biomarker panel as an adjunct to earlier diagnosis and initiation of treatment.

## Author Contributions

Dr. YL designed the study, developed the dimensional gradient reduction statistical approach, analyzed and interpreted the data, and drafted and revised the manuscript. Dr. RY and Dr. DN designed the schizophrenia case-control study, provided expertise in epidemiology, interpreted the data analysis, and drafted and revised the manuscript. Dr. MB interpreted the results and revised the manuscript. Dr. DC and Dr. TL interpreted the data analysis. Dr. DC also provided expertise in epidemiology. All authors read and approved the final manuscript.

## Conflict of Interest Statement

The research was conducted in the absence of any commercial or financial relationships that could be construed as a potential conflict of interest. The views expressed are the authors’ alone and do not represent the opinion of the Walter Reed Army Institute of Research (WRAIR), the Uniformed Services University of the Health Sciences (USUHS), and the Department of the Army or the Department of Defense.
